# Prognostic value of immunotherapy in advanced NSCLC based on baseline and dynamic changes in HALP

**DOI:** 10.17305/bb.2024.10833

**Published:** 2024-07-14

**Authors:** Hui Su, Chao Yu, Guiming Sun, Baozhong Wang, Yingjie Gao, Xiaolan Liu, Qingcui Song, Xuezhen Ma

**Affiliations:** 1 Department of Oncology, Medical College of Qingdao University, Qingdao, China; 2 Department of Oncology, Liaocheng People’s Hospital, Liaocheng, China; 3 Department of Orthopedics, Liaocheng People’s Hospital, Liaocheng, China; 4 Clinical Medicine, Shandong Second Medical University, Weifang, China; 5 Department of Oncology, Affiliated Qingdao Central Hospital of Qingdao University, Qingdao Cancer Hospital, Qingdao, China

**Keywords:** Non-small cell lung cancer (NSCLC), immune checkpoint inhibitors (ICIs), Hemoglobin–albumin–lymphocyte–platelet (HALP), Neutrophil to-lymphocyte ratio (NLR), Platelet-to-lymphocyte ratio (PLR), dynamics

## Abstract

Immune checkpoint inhibitors (ICIs) enhance the tumor-killing ability of T-cells in non-small cell lung cancer (NSCLC), improving overall survival (OS) and revolutionizing treatment for advanced stages. However, challenges remain, such as low response rates and the lack of effective markers for selecting candidates. This study evaluated the impact of hemoglobin, albumin, and platelet (HALP), neutrophil-to-lymphocyte ratio (NLR), and platelet-to-lymphocyte ratio (PLR) on the efficacy of immunotherapy and survival outcomes in advanced NSCLC. Additionally, it aimed to develop a nomogram based on these parameters. Clinical and hematological data from NSCLC patients who received immunotherapy were analyzed. Efficacy was assessed using the immune Response Evaluation Criteria in Solid Tumors (iRECIST), and progression-free survival (PFS) and OS were evaluated. Prediction models incorporated baseline and post-treatment HALP, NLR, and PLR values. The 203 patients had a median follow-up of 16 months, a median PFS (mPFS) of seven months (6.0–8.0), while the median OS (mOS) was not reached (24.0–not available). Pretreatment PLR (PLR0) was associated with a higher disease control rate (DCR) (odds ratio [OR] ═ 0.258), while initial immunotherapy and NLR after four treatment cycles (NLR4C) significantly improved the objective response rate (ORR). Cox regression analysis showed that pretreatment HALP (HALP0), HALP after four cycles of treatment (HALP4C), and pretreatment NLR (NLR0) significantly impacted PFS. Additionally, HALP0, NLR0, and PLR after four treatment cycles (PLR4C) were associated with OS. The C-indices for PFS and OS were 0.823 and 0.878, respectively, indicating good predictive accuracy. HALP, NLR, and PLR at various time points effectively predicted immunotherapy response in advanced NSCLC patients, with low HALP combined with high NLR and PLR indicating a poor prognosis. These findings could serve as the basis for stratified randomized controlled trials (RCTs) in the future.

## Introduction

According to the latest estimates from the International Agency for Research on Cancer, nearly 20 million new cancer cases were diagnosed in 2022, including non-melanoma skin cancers (NMSCs) [1]. Lung cancer was the most frequently diagnosed, accounting for approximately 2.5 million new cases (12.4% of all cancers worldwide). It also led to the most cancer-related deaths, with an estimated 1.8 million fatalities (18.7%) [1]. Most lung cancer patients are diagnosed at an advanced stage, and the 5-year survival rate for advanced-stage lung cancer is only 19% [2]. However, the development of immunotherapies has somewhat improved the prognosis for patients with non-small cell lung cancer (NSCLC) [3]. Immunotherapy, particularly, the use of immune checkpoint inhibitors (ICIs) targeting programmed cell death 1 (PD-1) and its ligand PD-L1, has become a key treatment for driver-negative advanced NSCLC [4–7]. Despite its effectiveness, immunotherapy can lead to serious immune-related adverse effects, making accurate biomarkers essential for determining which patients will benefit most. PD-L1 expression and tumor mutation burden (TMB) are currently the most widely used biomarkers to predict the efficacy of NSCLC immunotherapy [8]. However, challenges remain in detecting PD-L1 and TMB. For instance, the platforms, scoring systems, and interpretation criteria for PD-L1 vary, making it difficult to standardize results. Additionally, the heterogeneous and dynamic nature of PD-L1 expression complicates its accurate assessment. Even among patients with negative PD-L1 expression, 10%–20% may still respond to ICIs [9]. Methods for evaluating TMB, such as whole exome sequencing and targeted gene sequencing, are limited by complexity, cost, and time. Moreover, the FDA has not yet approved the use of TMB-targeted sequencing panels in routine clinical practice. As such, the application of these methods is largely confined to research settings. Therefore, easily accessible biomarkers are needed to better identify candidates for immunotherapy.

In recent years, several inflammatory markers have emerged as potential predictors in oncology, and numerous hematological inflammatory indicators have been shown to correlate with NSCLC prognosis. For example, the neutrophil-to-lymphocyte ratio (NLR), platelet-to-lymphocyte ratio (PLR), systemic immune-inflammation index (SII) [10], and prognostic nutritional index (PNI) have all been associated with malignancy and provide critical prognostic information [11–15]. Furthermore, the hemoglobin–albumin–lymphocyte–platelet (HALP) score has been linked to the prognosis of several cancers, including kidney [16], esophageal [17], pancreatic [18], small-cell lung [19, 20], bladder [21], and prostate cancer [22]. However, the clinical relevance of these values at baseline and at different time points following treatment is still debated. This study aims to explore the clinical significance of baseline and dynamic changes in HALP, NLR, and PLR values for predicting prognosis in NSCLC patients and to develop a nomogram to guide prognostic risk assessment.

## Materials and methods

### Clinical data

We retrospectively collected data from patients with advanced NSCLC who received ICIs between August 2019 and November 2022. Inclusion criteria were: (1) a pathological diagnosis of NSCLC, (2) clinical stage III (inoperable) or stage IV, (3) receipt of immunotherapy or combination therapy, and (4) complete medical and imaging records for efficacy evaluation. Patients were excluded if they had: (1) small-cell lung cancer, (2) autoimmune diseases, (3) symptomatic pulmonary interstitial disease or other severe comorbidities, or (4) hematologic disorders. A total of 203 patients met the criteria. Informed consent was waived due to the study’s retrospective and anonymous design.

Clinicopathological data for the patients included sex, age, pathological type, Eastern Cooperative Oncology Group Performance Status (ECOG-PS) score, tumor-node-metastasis (TNM) staging, driver gene mutation type, smoking and drinking history, treatment lines, and treatment regimens. Hematological parameters—hemoglobin, neutrophils, albumin, lymphocytes, and platelets—were measured before treatment and after two and four cycles of therapy.

### Treatment regimens

Patients received 200-mg doses of pembrolizumab, tislelizumab, camrelizumab, sintilimab, or 240-mg toripalimab intravenously every three weeks. Combination regimens included immunotherapy plus chemotherapy or anti-angiogenic agents. Immunotherapy was administered as a subsequent-line treatment for patients with EGFR mutations. Definitions: HALP ═ hemoglobin (g/L) × albumin (g/L) × lymphocytes (10^9^/L) / platelets (10^9^/L); NLR ═ neutrophil count (10^9^/L) / lymphocyte count (10^9^/L); PLR ═ platelet count (10^9^/L) / lymphocyte count (10^9^/L).

**Table 1:** Baseline characteristics of the patients

**Characteristics**		***n***	**Percentage (%)**
Sex	Male	158	77.83
	Female	45	22.17
Age	<65	99	48.77
	≥65	104	51.23
Smoking	No	75	36.95
	Yes	128	63.05
Drinking	No	129	63.55
	Yes	74	36.45
ECOG-PS	0–1	186	91.63
	2	17	8.37
Histology	Non-squamous carcinoma	110	54.19
	Squamous carcinoma	93	45.81
TNM stage	III	87	42.86
	IV	116	57.14
Gene mutation	No	49	24.14
	Yes	48	23.64
	Unknown	106	52.22
Option of treatment	Monotherapy	83	40.89
	Combination therapy	120	59.11
Lines of treatment	1	125	61.58
	≥2	78	38.42

ECOG-PS: Eastern Cooperative Oncology Group Performance Status; TNM stage: Tumor node metastasis stage.

### Follow-up and assessment

Patients were followed up via outpatient or inpatient visits and phone calls. Treatment responses, recurrence, and death times were recorded. The immune Response Evaluation Criteria in Solid Tumors (iRECIST) criteria were used to evaluate treatment response. Complete response (CR), partial response (PR), stable disease (SD), and progressive disease (PD) were measured. The objective response rate (ORR) was defined as CR + PR, while the disease control rate (DCR) was CR + PR + SD. Progression-free survival (PFS) was the time from immunotherapy initiation to disease progression or death. Overall survival (OS) was the time from disease onset to death from any cause or the last follow-up, whichever came first.

### Ethical statement

This study was approved by the Ethical Committee of Liaocheng People’s Hospital (approval number: 2023246). The requirement for written informed consent was waived because of the retrospective study design.

### Statistical analysis

IBM SPSS, X-tile, and R software were used for statistical analysis and visualization. X-tile was used to determine the optimal cutoff values for HALP, NLR, and PLR [23]. Chi-square tests and logistic regression were used to identify variables significantly influencing ORR and DCR. Kaplan–Meier survival analysis and log-rank tests compared OS and PFS among different groups. Cox regression analysis was used to assess prognostic factors, and a nomogram was constructed based on independent predictors. Bootstrap sampling was used to verify the nomogram and 1000 bootstrap resamples were applied to reduce overfitting using the **rms** package. Bootstrapping is a nonparametric technique that generates new datasets by repeatedly drawing random samples with replacement from the original dataset. This method helps create more robust predictions. The C-index and calibration curves were used to evaluate the model’s accuracy. All statistical tests were two-sided, with significance set at *P* < 0.05.

## Results

**Figure 1.**
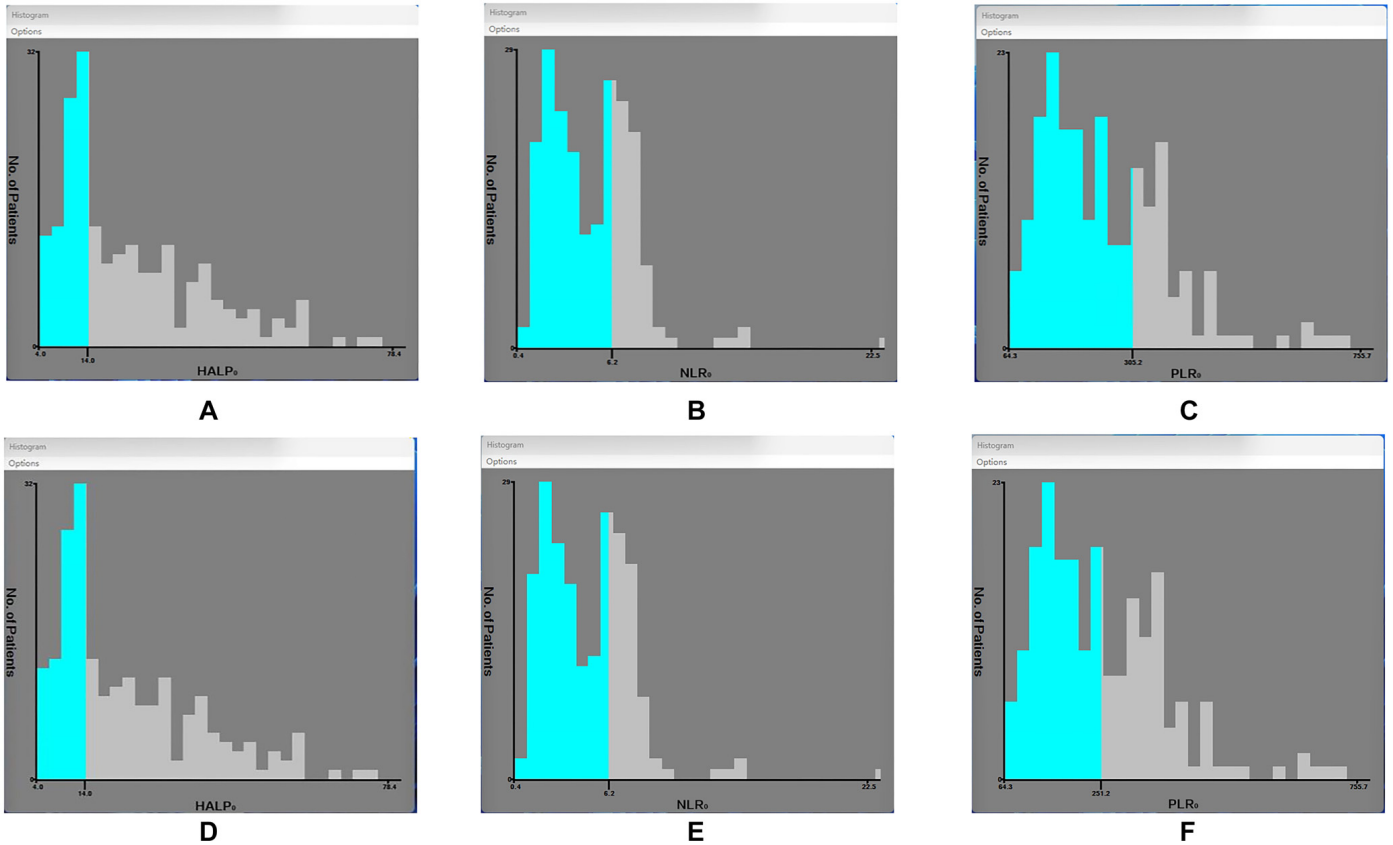
**The cutoff points for NLR0/PLR0/HAPL_0_ of PFS and OS using the X-tile program**. (A) HALP0 of PFS; (B) NLR0 of PFS; (C) PLR0 of PFS; (D) HALP0 of OS; (E) NLR0 of OS; (F) PLR0 of OS. OS: Overall survival; PFS: Progression-free survival; HALP0: Hemoglobin, albumin, and platelet before treatment; NLR0: Neutrophil-to-lymphocyte ratio before treatment; PLR0: Platelet-to-lymphocyte ratio before treatment.

### Clinical characteristics of the patients

A total of 203 patients with advanced NSCLC were included in this study. The average age at diagnosis was 64.3 years. Most patients were male (77.83%, *n* ═ 158) and had an ECOG-PS score of 0–1 (91.63%, *n* ═ 186). The histological type was divided into two groups: 45.81% had squamous cell carcinoma, while 54.19% had non-squamous cell carcinoma. In terms of disease stage, 42.86% had stage III NSCLC, and 57.14% had stage IV disease. Mutations in genes such as EGFR, KRAS, ALK, ROS-1, and BRAF were detected in 23.64% of cases (*n* ═ 48), while 52.22% of cases (*n* ═ 106) had no mutations. Additionally, 40.89% of patients (*n* ═ 83) were treated with monotherapy, and 59.11% (*n* ═ 120) were treated with combination therapy. First-line therapy was given to 61.58% of patients (*n* ═ 125), and 38.42% (*n* ═ 78) received second-line or later therapies. Full details are shown in Table 1.

### Optimal cutoff

X-tile software was used to determine the optimal cutoff values for HALP, NLR, and PLR in predicting PFS and OS. The optimal cutoff values were 13.99 for HALP, 6.23 for NLR, and 305.21 for PLR in predicting PFS (Figure 1A–1C). For OS, the optimal cutoff values were also 13.99 for HALP, 6.23 for NLR, but 251.19 for PLR (Figure 1D–1F). Based on these cutoff points, patients were categorized into groups.

**Table 2:** Response to treatment

**Clinical indices**	**Disease control rate**			**Overall response rate**		
	***n***	**Percentage**	***P* value**	***n***	**Percentage**	***P* value**
*Sex*						
Male	136/158	86.08%	0.919	84/158	53.16%	0.777
Female	39/45	86.67%		25/45	55.56%	
*Age*						
<65	89/99	89.90%	0.137	48/99	48.48%	0.146
≥65	86/104	82.69%		61/104	58.65%	
*Smoking*						
No	67/75	89.33%	0.323	45/75	60.00%	0.168
Yes	108/128	84.38%		64/128	50.00%	
*Drinking*						
No	113/129	87.60%	0.448	68/129	52.71%	0.711
Yes	62/74	83.78%		41/74	55.41%	
*ECOG-PS*						
0-1	162/186	87.10%	0.224	96/186	51.61%	0.049
2	13/17	76.47%		13/17	76.47%	
*Histology*						
Non-squamous carcinoma	92/110	83.64%	0.248	66/110	60.00%	0.050
Squamous carcinoma	83/93	89.25%		43/93	46.24%	
*TNM stage*						
III	73/87	83.91%	0.411	48/87	55.17%	0.715
IV	102/116	87.93%		61/116	52.59%	
*Gene mutation*						
No	41/49	83.67%	0.780	25/49	51.02%	0.745
Yes	41/48	85.42%		28/48	58.33%	
Unknow	93/106	87.74%		56/106	52.83%	
*Option of treatment*						
Monotherapy	73/83	87.95%	0.549	38/83	45.78%	0.060
Combination therapy	102/120	85%		71/120	59.17%	
*Lines of treatment*						
1	109/125	87.20%	0.603	56/125	44.80%	0.001
≥2	66/78	84.62%		53/78	67.95%	
*HALP0 (PFS and OS)*						
≤13.99	55/82	67.07%	<0.001	57/82	69.51%	<0.001
>13.99	120/121	99.17%		52/121	42.98%	
*HALP2C (PFS and OS)*						
≤13.99	21/32	65.63%	<0.001	21/32	65.63%	0.140
>13.99	154/171	90.06%		88/171	51.46%	
*HALP4C (PFS and OS)*						
≤13.99	20/34	58.82%	<0.001	24/34	70.59%	0.030
>13.99	155/169	91.72%		85/169	50.30%	
*NLR0 (PFS and OS)*						
≤6.23	120/122	98.36%	<0.001	52/122	42.62%	<0.001
>6.23	55/81	67.90%		57/81	70.37%	
*NLR2C (PFS and OS)*						
≤6.23	164/187	87.70%	0.035	98/187	52.41%	0.208
>6.23	11/16	68.75%		11/16	68.75%	
*NLR4C (PFS and OS)*						
≤6.23	160/177	90.40%	<0.001	88/177	49.72%	0.003
>6.23	15/26	57.69%		21/26	80.77%	
*PLR0 (PFS)*						
≤305.21	133/137	97.08%	<0.001	67/137	48.91%	0.049
>305.21	42/66	63.64%		42/66	63.64%	
*PLR2C (PFS)*						
≤305.21	152/173	87.86%	0.101	90/173	52.02%	0.251
>305.21	23/30	76.67%		19/30	63.33%	
*PLR4C (PFS)*						
≤305.21	156/174	89.66%	<0.001	89/174	51.15%	0.075
>305.21	19/29	65.52%		20/29	68.97%	
*PLR0 (OS)*						
≤251.19	115/116	99.14%	<0.001	53/116	45.69%	0.008
>251.19	60/87	68.97%		56/87	64.37%	
*PLR2C (OS)*						
≤251.19	135/153	88.24%	0.143	81/153	52.94%	0.706
>251.19	40/50	80.00%		28/50	56.00%	
*PLR4C (OS)*						
≤251.19	136/150	90.67%	0.002	76/150	50.67%	0.146
>251.19	39/53	73.58%		33/53	62.26%	

ECOG-PS: Eastern Cooperative Oncology Group Performance Status; TNM stage: Tumor node metastasis stage; PFS: Progression-free survival; OS: Overall survival; HALP0: Hemoglobin, albumin, and platelet before treatment; HALP2C: Hemoglobin, albumin, and platelet after two cycles of treatment; HALP4C: Hemoglobin, albumin, and platelet after four cycles of treatment; NLR0: Neutrophil-to-lymphocyte ratio before treatment; NLR2C: Neutrophil-to-lymphocyte ratio after two cycles of treatment; NLR4C: Neutrophil-to-lymphocyte ratio after four cycles of treatment; PLR0: Platelet-to-lymphocyte ratio before treatment; PLR2C: Platelet-to-lymphocyte ratio after two cycles of treatment; PLR4C: Platelet-to-lymphocyte ratio after four cycles of treatment.

### Assessment

The chi-square test revealed that baseline HALP, NLR, and PLR values, as well as changes after two and four cycles of treatment, influenced the DCR. Factors, such as ECOG-PS score, number of immunotherapy lines, baseline HALP and NLR values, and changes in these markers after four cycles were significantly correlated with ORR. Multivariate logistic regression analysis showed that baseline PLR ≤ 305.21 was associated with a higher DCR (OR ═ 0.258, 95% CI: 0.070–0.946), while initial immunotherapy (OR ═ 2.697, 95% CI: 1.430–5.089) and NLR after four cycles (OR ═ 4.273, 95% CI: 1.039–17.582) were associated with higher ORR (Table 2).

### Survival analysis

**Figure 2.**
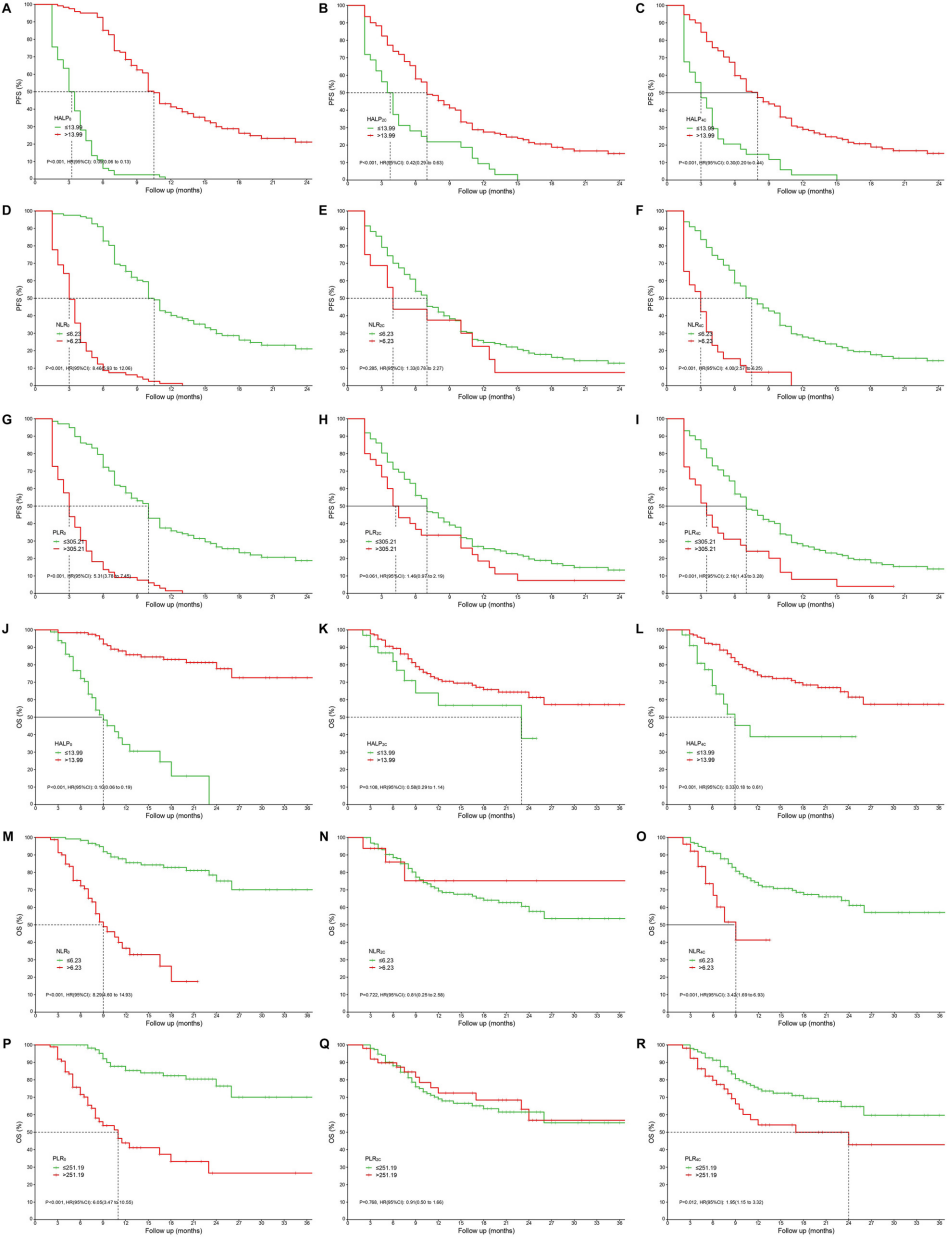
**Kaplan–Meier curves for PFS and OS.** (A and J) PFS and OS stratified by the baseline HALP0 index; (B and K) PFS and OS stratified by the baseline HALP2C index; (C and L) PFS and OS stratified by the baseline HALP4C index; (D and M) PFS and OS stratified by the baseline NLR0 index; (E and N) PFS and OS stratified by the baseline NLR2C index; (F and O) PFS and OS stratified by the baseline NLR4C index; (Gand P) PFS and OS stratified by the baseline PLR0 index; (H and Q) PFS and OS stratified by the baseline PLR2C index; (I and R) PFS and OS stratified by the baseline PLR4C index. PFS: Progression-free survival, OS: Overall survival; HALP0: Hemoglobin, albumin, and platelet before treatment; HALP2C: Hemoglobin, albumin, and platelet after two cycles of treatment; HALP4C: Hemoglobin, albumin, and platelet after four cycles of treatment; NLR0: Neutrophil-to-lymphocyte ratio before treatment; NLR2C: Neutrophil-to-lymphocyte ratio after two cycles of treatment; NLR4C: Neutrophil-to-lymphocyte ratio after four cycles of treatment; PLR0: Platelet-to-lymphocyte ratio before treatment; PLR2C: Platelet-to-lymphocyte ratio after two cycles of treatment; PLR4C: Platelet-to-lymphocyte ratio after four cycles of treatment.

Kaplan–Meier survival analysis and log-rank tests showed that low HALP and high NLR and PLR values were associated with shorter PFS and OS (Figure 2A–2R). Specifically, low baseline HALP and its changes after treatment (HALP2C, HALP4C) and high baseline NLR and PLR were significantly associated with shorter PFS. Similar trends were observed for OS, where low HALP and high NLR and PLR values predicted worse survival outcomes.

Univariate Cox analysis identified age, TNM stage, treatment regimen, HALP, NLR, and PLR as significant factors affecting PFS and OS. Multivariate Cox analysis showed that patients aged ≥65 years (HR ═ 2.05, 95% CI: 1.48–2.84, *P* < 0.001), HALP0 ≤ 13.99 (HR ═ 0.19, 95% CI: 0.11–0.32, *P* < 0.001), HALP4C ≤ 13.99 (HR ═ 0.38, 95% CI: 0.18–0.79, *P* ═ 0.01), and NLR0 > 6.23 (HR ═ 5.11, 95% CI: 3.05–8.55, *P* < 0.001) had a higher risk of disease progression. The TNM stage was also an independent predictor of PFS (HR ═ 1.62, 95% CI: 1.16–2.25, *P* ═ 0.004), with stage IV patients facing a higher risk of progression compared to stage III patients (Table 3).

For OS, multivariate Cox analysis showed that age ≥65 years (HR ═ 3.17, 95% CI: 1.80–5.60, *P* < 0.001), HALP0 ≤ 13.99 (HR ═ 0.34, 95% CI: 0.15–0.80, *P* ═ 0.013), NLR0 > 6.23 (HR ═ 2.99, 95% CI: 1.27–7.01, *P* ═ 0.012), and PLR4C > 251.19 (HR ═ 3.00, 95% CI: 1.67–5.40, *P* < 0.001) significantly increased the risk of death. TNM stage (HR ═ 2.22, 95% CI: 1.18–4.19, *P* ═ 0.013) and treatment regimen (HR ═ 0.24, 95% CI: 0.13–0.47, *P* < 0.001) were also independent predictors of OS (Table 4).

**Table 3:** Univariate and multivariate analysis of PFS

**Clinical indexes**	**Univariate analysis**		**Multivariate analysis**	
	**HR (95% CI)**	***P* value**	**HR (95% CI)**	***P* value**
*Sex*				
Male				
Female	1.06 (0.74, 1.51)	0.752		
*Age*				
<65				
≥65	1.40 (1.03, 1.91)	0.029	2.05 (1.48, 2.84)	<0.001
*Smoking*				
No				
Yes	1.07 (0.78, 1.46)	0.673		
*Drinking*				
No				
Yes	0.91 (0.67, 1.25)	0.566		
*ECOG-PS*				
0-1				
2	0.80 (0.44, 1.49)	0.487		
*Histology*				
Non-squamous carcinoma				
Squamous carcinoma	0.91 (0.67, 1.23)	0.545		
*TNM stage*				
III				
IV	1.47 (1.08, 2.01)	0.014	1.62 (1.16, 2.25)	0.004
*Gene mutation*				
No				
Yes	0.89 (0.58, 1.37)	0.604		
Unknow	0.88 (0.61, 1.26)	0.478		
*Option of treatment*				
Monotherapy				
Combination therapy	0.55 (0.41, 0.75)	<0.001		
*Lines of treatment*				
1				
≥2	1.13 (0.83, 1.54)	0.427		
*HALP0*				
≤13.99				
>13.99	0.09 (0.06, 0.13)	<0.001	0.19 (0.11, 0.32)	<0.001
*HALP2C*				
≤13.99				
>13.99	0.42 (0.29, 0.63)	<0.001	1.56 (0.97, 2.51)	0.066
*HALP4C*				
≤13.99				
>13.99	0.30 (0.20, 0.44)	<0.001	0.38 (0.18, 0.79)	0.010
*NLR0*				
≤6.23				
>6.23	8.46 (5.93, 12.06)	<0.001	5.11 (3.05, 8.55)	<0.001
*NLR2C*				
≤6.23				
>6.23	1.33 (0.78, 2.27)	0.295		
*NLR4C*				
≤6.23				
>6.23	4.00 (2.57, 6.25)	<0.001		
*PLR0*				
≤305.21				
>305.21	5.31 (3.78, 7.45)	<0.001		
*PLR2C*				
≤305.21				
>305.21	1.46 (0.97, 2.19)	0.070		
*PLR4C*				
≤305.21				
>305.21	2.16 (1.43, 3.28)	<0.001	0.54 (0.25, 1.18)	0.120

ECOG-PS: Eastern Cooperative Oncology Group Performance Status; TNM stage: Tumor node metastasis stage; HALP0: Hemoglobin, albumin, and platelet before treatment; HALP2C: Hemoglobin, albumin, and platelet after two cycles of treatment; HALP4C: Hemoglobin, albumin, and platelet after four cycles of treatment; NLR0: Neutrophil-to-lymphocyte ratio before treatment; NLR2C: Neutrophil-to-lymphocyte ratio after two cycles of treatment; NLR4C: Neutrophil-to-lymphocyte ratio after four cycles of treatment; PLR0: Platelet-to-lymphocyte ratio before treatment; PLR2C: Platelet-to-lymphocyte ratio after two cycles of treatment; PLR4C: Platelet-to-lymphocyte ratio after four cycles of treatment; PFS: Progression-free survival.

**Table 4:** Univariate and multivariate analysis of OS

**Clinical indexes**	**Univariate analysis**		**Multivariate analysis**	
	**HR (95% CI)**	***P* value**	**HR (95% CI)**	***P* value**
*Sex*				
Male				
Female	0.77 (0.40, 1.49)	0.440		
*Age*				
<65				
≥65	2.19 (1.28, 3.74)	0.004	3.17 (1.80, 5.60)	<0.001
*Smoking*				
No				
Yes	1.50 (0.86, 2.60)	0.153		
*Drinking*				
No				
Yes	1.32 (0.79, 2.21)	0.284		
*ECOG-PS*				
0-1				
2	1.85 (0.84, 4.08)	0.127		
*Histology*				
Non-squamous carcinoma				
Squamous carcinoma	0.63 (0.37, 1.07)	0.084		
*TNM stage*				
III				
IV	2.81 (1.54, 5.11)	0.001	2.22 (1.18, 4.19)	0.013
*Gene mutation*				
No				
Yes	0.75 (0.37, 1.52)	0.421		
Unknown	0.76 (0.42, 1.37)	0.367		
*Option of treatment*				
Monotherapy				
Combination therapy	0.18 (0.10, 0.33)	<0.001	0.24 (0.13, 0.47)	<0.001
*Lines of treatment*				
1				
≥2	1.03 (0.61, 1.74)	0.903		
*HALP0*				
≤13.99				
>13.99	0.10 (0.06, 0.19)	<0.001	0.34 (0.15, 0.80)	0.013
*HALP2C*				
≤13.99				
>13.99	0.58 (0.29, 1.14)	0.115		
*HALP4C*				
≤13.99				
>13.99	0.33 (0.18, 0.61)	<0.001		
*NLR0*				
≤6.23				
>6.23	8.29 (4.60, 14.93)	<0.001	2.99 (1.27, 7.01)	0.012
*NLR2C*				
≤6.23				
>6.23	0.81 (0.25, 2.58)	0.719		
*NLR4C*				
≤6.23				
>6.23	3.42 (1.69, 6.93)	0.001		
*PLR0*				
≤251.19				
>251.19	6.05 (3.47, 10.55)	<0.001		
*PLR2C*				
≤251.19				
>251.19	0.91 (0.50, 1.66)	0.763		
*PLR4C*				
≤251.19				
>251.19	1.95 (1.15, 3.32)	0.014	3.00 (1.67, 5.40)	<0.001

ECOG-PS: Eastern Cooperative Oncology Group Performance Status; TNM stage: Tumor node metastasis stage; HALP0: Hemoglobin, albumin, and platelet before treatment; HALP2C: Hemoglobin, albumin, and platelet after two cycles of treatment; HALP4C: Hemoglobin, albumin, and platelet after four cycles of treatment; NLR0: Neutrophil-to-lymphocyte ratio before treatment; NLR2C: Neutrophil-to-lymphocyte ratio after two cycles of treatment; NLR4C: Neutrophil-to-lymphocyte ratio after four cycles of treatment; PLR0: Platelet-to-lymphocyte ratio before treatment; PLR2C: Platelet-to-lymphocyte ratio after two cycles of treatment; PLR4C: Platelet-to-lymphocyte ratio after four cycles of treatment.

### Establishment of a nomogram and predictive models

**Figure 3.**
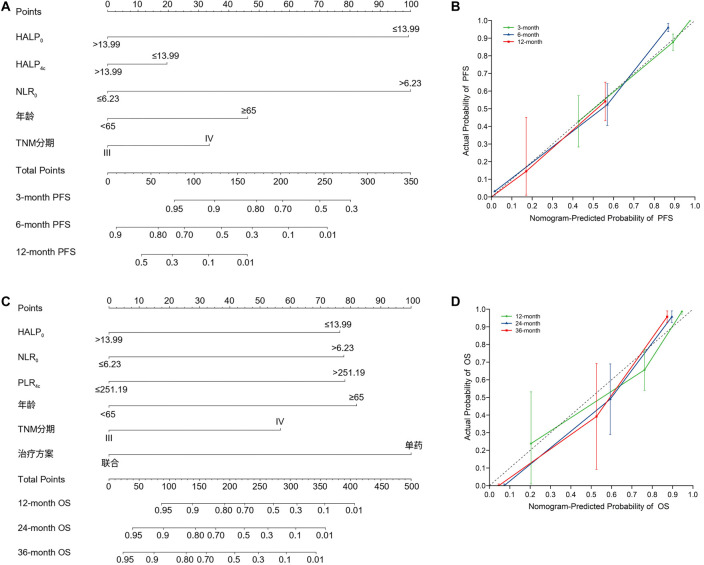
**Nomogram predicting the PFS and OS and calibration plots.** (A) Nomogram predicting the PFS; (B) Calibration curve for predicting the probability of 3-month, 6-month, and 12-month PFS; (C) Nomogram predicting the OS; (D) Calibration curve for predicting the probability of 12-month, 24-month, and 36-month OS. OS: Overall survival; PFS: Progression-free survival.

Based on the results of the Cox multivariate analysis, we constructed a nomogram to predict PFS and OS. The C-index of the PFS prediction model was 0.823 (95% CI: 0.799–0.848), and for OS, it was 0.878 (95% CI: 0.845–0.912), indicating high predictive accuracy (Figure 3). Bootstrap sampling (1000 resamples) was used for internal validation, confirming the robustness of the model. Calibration curves demonstrated good agreement between predicted and actual outcomes.

## Discussion

ICIs are highly effective for patients with advanced NSCLC and a PD-L1 tumor proportion score (TPS) ≥ 50%, outperforming conventional chemotherapy. Moreover, several studies on ICI therapy for NSCLC have demonstrated significant benefits regardless of PD-L1 expression status [4–7], with fewer side effects and lower financial burden for patients. However, efficient, affordable, and accessible markers are needed to identify patients who might benefit from ICI therapy. The HALPNLR and PLR values are calculated from basic blood tests, making them cost-effective and easy to obtain. Additionally, we developed a nomogram as a simple, practical tool for predicting survival, identifying high-risk patients, and facilitating early interventions that can improve quality of life. Our study showed that HALP, NLR, and PLR values at various time points effectively predicted immunotherapy response in advanced NSCLC patients, where low HALP combined with high NLR and PLR values was associated with poor prognosis. Therefore, our data could serve as a reference for patient stratification in future randomized controlled trials (RCTs) for NSCLC and other related diseases.

Research indicates that the development, progression, and metastasis of malignant tumors are closely linked to the body’s nutritional, inflammatory, and immune status [24]. A low HALP value could be due to low hemoglobin, low lymphocytes, or high platelets, while a high NLR is typically associated with high neutrophil or low lymphocyte counts, and a high PLR is due to high platelet or low lymphocyte counts. Many advanced cancer patients experience some degree of anemia, increasing their risk of mortality from various tumors [25]. Neutrophils are the body’s first line of defense against inflammation and infection and are key to the complex relationship between inflammation and tumor growth, either inhibiting or promoting cancer [26, 27]. Blood albumin levels also reflect the body’s nutritional state and, as a negative acute-phase protein, can indicate inflammation [28]. Platelets release cytokines, such as transforming growth factor-β1 and vascular endothelial growth factor, which contribute to tumor growth, metastasis, angiogenesis, and immune escape [29]. Lymphocytes play a central role in the immune system, initiating a cytotoxic response to prevent tumor cell proliferation, invasion, and metastasis [30]. Therefore, low HALP combined with high NLR and PLR could be a reliable biomarker for tumor progression and poor prognosis. Compared with single markers, a composite index involving HALP, NLR, and PLR integrates multiple parameters, offering a more comprehensive view of the inflammatory, immune, and nutritional status. Composite indicators have already been applied to assess the prognosis of various cancers. To our knowledge, this is the first study to examine the prognostic value of baseline and dynamic changes in multiple inflammatory markers for predicting responses to full-line immunotherapy in NSCLC.

In this retrospective study, we evaluated the clinical characteristics and prognosis of 203 NSCLC patients, forecasting clinical outcomes by analyzing peripheral blood markers of inflammation and nutrition potentially linked to the disease. Unlike previous studies, this work developed several models using HALP, NLR, and PLR data at different time points (0, 2 cycles, 4 cycles) to dynamically predict treatment efficacy and survival. While an earlier study [31] focused only on patients receiving first-line immunotherapy, our study included patients across all lines of immunotherapy, including those beyond the first line. The earlier study also included only patients with wild-type EGFR, ALK, and ROS-1 genes, whereas we included patients with both mutated and wild-type genes. Patients with gene mutations were given targeted therapy as first-line treatment, with immunotherapy used as a subsequent line. Additionally, the previous study [31] grouped patients using the interquartile range, which could introduce greater variability, while we used the X-tile program to determine optimal cutoffs. Interestingly, we found that HALP and NLR had identical optimal cutoffs for both PFS and OS (HALP ═ 13.99, NLR ═ 6.23), whereas PLR had different cutoffs for OS (PLR ═ 251.19) and PFS (PLR ═ 305.21), suggesting our algorithm is more robust. Further, our data indicate that HALP0 > 13.99 and NLR0 ≤ 6.23 are associated with longer PFS and OS, HALP4C > 13.99 correlates with longer PFS, and PLR4C ≤ 251.19 correlates with longer OS.

NLR and PLR are general markers of the immune response to stress conditions [32]. A review [33] summarized various studies confirming that inflammatory biomarkers can predict clinical outcomes in NSCLC patients treated with ICIs. Yuan et al. [34] demonstrated that low baseline NLR and PLR are significantly associated with better PFS (*P* ═ 0.011 and 0.027, respectively) and OS (*P* ═ 0.042 and 0.039, respectively) in advanced NSCLC patients treated with ICIs. Chen et al. [35] observed that NSCLC patients with a baseline NLR ≤ 4 treated with immunotherapy had improved PFS (5.7 vs 2.0 months, *P* ═ 0.0083) and OS (21.3 vs. 5.0 months, *P* ═ 0.0163). Their findings align with ours, showing that even patients with EGFR-sensitive mutations can benefit from anti-PD-1 inhibitors after progressing on EGFR-TKIs. However, most studies only focus on baseline data. Asano et al. [36] highlighted that dynamic changes in NLR and PNI were independent predictors of both best objective response and OS, serving as useful biomarkers in ICI-treated NSCLC patients with bone metastases. In contrast, our study did not stratify patients with bone metastases separately. Olgun et al. [37] reported that high post-treatment NLR ≥ 5 (*P* ═ 0.004) and PLR ≥ 170 (*P* ≤ 0.001) were independent prognostic factors for shorter OS, which is consistent with our findings from dynamic observations of these markers. These conclusions also apply to small cell lung cancer (SCLC) [38].

The HALP score has been shown to correlate with prognosis in various cancers, but studies specifically examining HALP in NSCLC are limited. In 2022, Wei et al. [39] performed a retrospective analysis of 362 NSCLC patients receiving adjuvant chemotherapy and found that a HALP score below 48.2 (determined using X-tile) was linked to poorer OS (*P* ═ 0.02) and DFS (*P* < 0.01). However, that study included patients receiving adjuvant chemotherapy, unlike ours. Fang et al. [31] examined inoperable NSCLC patients undergoing first-line immunotherapy plus chemotherapy and found that HALP did not significantly predict PFS (*P* ═ 0.771) or OS (*P* ═ 0.996), which differed from our findings. High pretreatment PLR (OR ═ 2.612) and increased NLR during follow-up (OR ═ 2.516) were strongly linked to a lower ORR. Further, high pretreatment PLR (HR ═ 2.319) predicted shorter PFS, while high pretreatment NLR (HR ═ 1.635) and increased NLR (HR ═ 1.663) and PLR (HR ═ 1.691) predicted shorter OS.

To assess the accuracy of our predictive model, we developed a nomogram, calculated the C-index, and plotted calibration curves. Internal validation showed that the C-index for PFS and OS was 0.823 (95% CI: 0.799–0.848) and 0.878 (95% CI: 0.845–0.912), respectively, indicating high predictive accuracy.

### Study limitations

This study has several limitations. First, it was a single-center, retrospective study with a limited sample size, which may affect the generalizability of the results. Multicenter studies with larger cohorts are needed to validate these findings. Second, the lack of a chemotherapy-only control group makes it difficult to differentiate the specific effects of immunotherapy on the hematological markers studied. Finally, there is no consensus on the optimal cutoff values for HALP, NLR, and PLR, which may vary depending on the study population and methodology. Future research should aim to establish standardized cutoff values for these markers.

## Conclusion

The HALP, NLR, and PLR values, both at baseline and during treatment, effectively predicted the response to immunotherapy in patients with advanced NSCLC. Low HALP combined with high NLR and PLR values indicated a poor prognosis. These markers, which are inexpensive and easy to obtain, could serve as useful tools for patient stratification in future RCTs and clinical practice.

## Data Availability

The raw data supporting the conclusions of this article will be made available by the authors without any undue reservation.
